# Hepatic expression of IL-8, IL-10, and CXCL-14 in drug-induced liver injury: correlation with histopathology and prognostic value

**DOI:** 10.3389/fphar.2026.1882357

**Published:** 2026-08-12

**Authors:** Diandian Hao, Jialin Du, Hui Wang, Yuze Song, Yulin Ren, Xiao-Yu Wen

**Affiliations:** Department of Hepatology, Center of Infectious Diseases and Pathogen Biology, The First Hospital of Jilin University, Changchun, Jilin, China

**Keywords:** CXCL-14, cytokines, drug-induced liver injury(DILI), histopathology(HPE), IL-10, IL-8

## Abstract

**Background:**

Drug-induced liver injury (DILI) lacks reliable tissue-based biomarkers for diagnosing disease severity and predicting outcomes. This study investigated the hepatic expression of interleukin-8 (IL-8), interleukin-10 (IL-10), and chemokine ligand-14 (CXCL-14) in DILI and evaluated their diagnostic and prognostic relevance.

**Methods:**

Forty biopsy-proven DILI patients and ten healthy controls were enrolled. Immunohistochemistry was performed to quantify hepatic IL-8, IL-10, and CXCL-14 expression. Clinical variables, inflammation grades, and fibrosis stages were analyzed. Correlation analysis, logistic regression, and ROC curves were applied to assess diagnostic value and prognosis.

**Results:**

Hepatic IL-8, IL-10, and CXCL-14 were significantly elevated in DILI patients compared with controls (*P* < 0.05). IL-8 and CXCL-14 levels varied with inflammation grade, and IL-8 also correlated with fibrosis. Spearman analysis showed positive correlations of IL-8, IL-10, and CXCL-14 with inflammation severity, while IL-8 and CXCL-14 were associated with fibrosis. CXCL-14 additionally correlated with AST and GGT. Multivariate analysis identified CXCL-14 as an independent predictor of unfavorable outcomes (OR = 1.769, 95% CI: 1.062–2.947, *P* = 0.029). ROC analysis demonstrated good prognostic performance (AUC = 0.848).

**Conclusion:**

IL-8, IL-10, and CXCL-14 were significantly upregulated in DILI liver tissue. The expression level of IL-8 was closely associated with hepatic inflammation and fibrosis and may serve as a potential indicator of disease severity. CXCL-14 was identified as an independent factor associated with clinical prognosis and demonstrated promising prognostic performance in this cohort. These findings suggest that tissue chemokine profiling may have potential value for risk stratification in DILI, although further validation in larger prospective studies is warranted.

## 1 Introduction

Drug-induced liver injury (DILI) refers to hepatocellular damage caused by medications, herbal products, dietary supplements, or their metabolites. It is a globally prevalent but diagnostically challenging clinical condition, characterized by diverse clinical manifestations and complex histopathological subtypes. In severe cases, DILI may progress to liver failure and even death. The epidemiology of DILI exhibits marked regional variation. In Western countries, the annual incidence is approximately 4.0 per 100,000 population (De Valle et al., 2006; Andrade et al., 2005), whereas in China, it reaches as high as 23.8 per 100,000, with herbal medicine-related DILI accounting for 27% of cases (Shen et al., 2019). This highlights the influence of cultural practices on drug usage and underscores the urgent need for stricter regulation of traditional Chinese medicine. The causative agents also vary by region: in Asia, DILI is commonly associated with herbal remedies and anti-tuberculosis drugs (Zhang et al., 2022), whereas acetaminophen and nonsteroidal anti-inflammatory drugs (NSAIDs) are more frequently implicated in Western populations.

The pathogenesis of DILI is multifactorial and involves mechanisms such as drug bioactivation (Zanger and Schwab, 2013; Yan et al., 2018), bile acid metabolism dysregulation (Krähenbühl et al., 1994; Tujios and Fontana, 2011; Fattinger et al., 2001; Morgan et al., 2010), polymorphisms in hepatic transporter genes (Yoshikado et al., 2011), and drug–drug interactions (Christians et al., 2002). According to the latest clinical guidelines from the European Association for the Study of the Liver (EASL) (Euro pean Association for the Study of the Liver, 2019), DILI can be categorized into three subtypes based on the R value: hepatocellular (R ≥ 5), cholestatic (R ≤ 2), and mixed (2 < R < 5).

Currently, the diagnosis of DILI remains a diagnosis of exclusion, requiring comprehensive assessment of medication history, clinical presentation, serum biochemical parameters (e.g., ALT, AST, ALP), imaging findings, and histopathological evidence. Recent studies suggest that cytokines play a pivotal role in the immune-inflammatory processes underlying DILI. For instance, interleukin-8 (IL-8) exacerbates liver injury by promoting neutrophil recruitment and activating the PI3K/Akt signaling pathway (Ma et al., 2022). Although IL-10 exerts anti-inflammatory effects (Bourdi et al., 2002), it may paradoxically contribute to disease progression under certain conditions, such as acute liver failure (Berry et al., 2010). CXCL-14, a newly identified chemokine, regulates immune cell trafficking and exhibits a unique expression profile in DILI, although its precise role and regulatory mechanisms remain poorly understood.

To comprehensively evaluate different aspects of the immune response in DILI, we selected IL-8, IL-10, and CXCL-14 as representative cytokines. IL-8 represents pro-inflammatory signaling associated with neutrophil recruitment and hepatic inflammation, whereas IL-10 is a key anti-inflammatory cytokine involved in immune regulation and tissue protection. CXCL-14 was included because of its emerging role in immune cell trafficking and tissue remodeling, as well as the limited data currently available regarding its involvement in DILI. The combined evaluation of these cytokines was expected to provide a more comprehensive understanding of the inflammatory and immunoregulatory processes underlying DILI.

The aberrant expression of cytokines suggests their potential utility in the diagnosis, disease stratification, and prognostic assessment of DILI. This study aims to investigate the hepatic expression of IL-8, IL-10, and CXCL-14 in patients with DILI and to explore their correlations with clinical and pathological features, thereby providing insight into DILI subtyping and therapeutic intervention strategies.

## 2 Materials and methods

### 2.1 Study population

**FIGURE 1:**
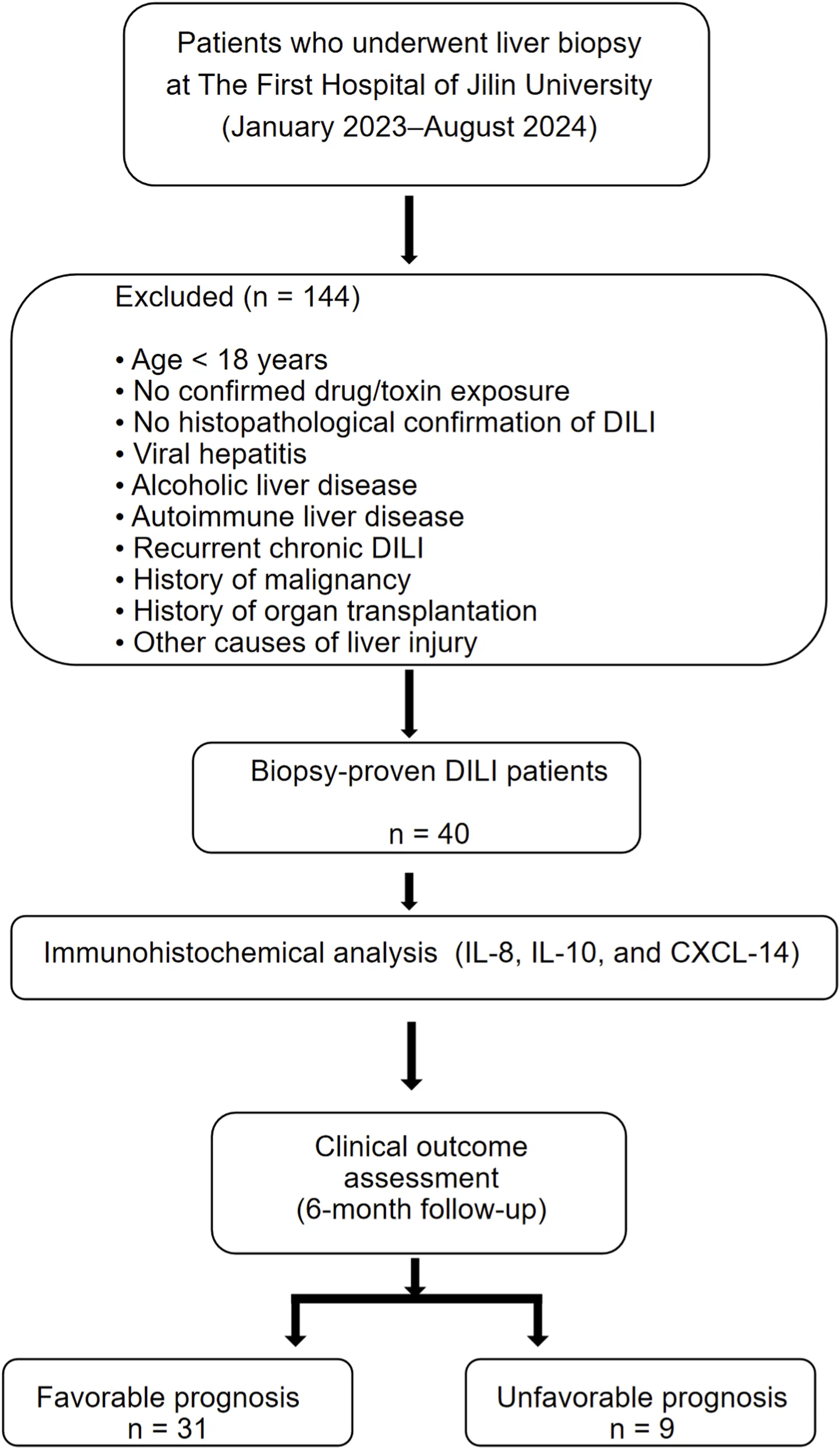
Flowchart of patient selection and study design. A total of 184 patients who underwent liver biopsy between January 2023 and August 2024 were screened. After application of the predefined inclusion and exclusion criteria, 40 biopsy-proven DILI patients were included in the study and underwent immunohistochemical analysis of IL-8, IL-10, and CXCL-14 expression. Clinical outcomes were assessed after 6 months of follow-up.

This retrospective study included 40 patients who were hospitalized in the Department of Hepatobiliary and Pancreatic Medicine at the First Hospital of Jilin University between January 2023 and August 2024 and were diagnosed with drug-induced liver injury (DILI) by liver biopsy. All patients had a documented history of drug use or exposure to hepatotoxic substances and met the diagnostic criteria outlined in the 2023 Chinese Guidelines for the Diagnosis and Treatment of Drug-Induced Liver Injury (Mao et al., 2024). In addition, causality assessment was retrospectively performed using the updated Roussel Uclaf Causality Assessment Method (RUCAM). All included patients achieved RUCAM scores ranging from 6 to 8, corresponding to a probable diagnosis of DILI. According to these guidelines, the diagnosis of DILI is based on a comprehensive assessment of drug exposure history, clinical manifestations, liver biochemical abnormalities, exclusion of alternative causes of liver injury, and histopathological evaluation when available. All DILI diagnoses were established by experienced hepatologists based on a comprehensive evaluation of medication exposure history, clinical presentation, laboratory findings, exclusion of competing liver diseases, and histopathological examination of liver biopsy specimens. The control group consisted of 10 healthy liver donors (7 males and 3 females; mean age, 42.5 years) who participated in the liver transplantation program at The First Hospital of Jilin University during the same study period (January 2023 to August 2024). These tissues were collected from surplus donor liver tissue during transplantation procedures. All donor tissues were collected with appropriate ethical approval and were anonymized before analysis. All donors had normal liver function tests, no history of liver disease, and no significant pathological abnormalities on histological examination. The study was approved by the Ethics Committee of the First Hospital of Jilin University and conducted in accordance with the Declaration of Helsinki. The patient selection process is summarized in Figure 1.

### 2.2 Inclusion criteria

1) Age ≥ 18 years; 2) A confirmed history of drug or toxin exposure; 3) Histopathological confirmation of DILI by liver biopsy.

### 2.3 Exclusion criteria

1) Evidence of other liver diseases such as viral hepatitis, alcoholic liver disease, or autoimmune liver disease; 2)Recurrent cases of chronic DILI rather than first-time onset; 3)History of malignancy or organ transplantation.

### 2.4 Data collection

Demographic characteristics, biochemical parameters, and liver histopathology reports were collected for all 40 DILI patients. Information on treatment and clinical outcomes was also recorded for further analysis.

#### 2.4.1 Histopathological grading

All patients underwent liver biopsy under ultrasound guidance using a 16G core needle after providing written informed consent. Tissue specimens with a length of ≥1.5 cm were fixed in 10% neutral buffered formalin, embedded in paraffin, sectioned, and stained with hematoxylin and eosin (H&E) using standard protocols. The degree of inflammatory activity was graded from G0 to G4 according to the Chinese pathological criteria for liver diseases. Fibrosis staging was classified from S0 to S4 based on the extent of fibrous deposition in the portal area and lobular septa. Histopathological grading and staging were determined according to the formal pathology reports and independently evaluated by two experienced pathologists.

#### 2.4.2 Immunohistochemical (IHC) staining

Primary antibodies against IL-8 (CST, 94407), IL-10 (Proteintech, 60269), and CXCL-14 (Proteintech, 10468) were used at a dilution of 1:200. The standard streptavidin-peroxidase (SP) three-step method was applied for immunohistochemical staining. For each specimen, five high-power field (HPF) images were selected. ImageJ software was used to calculate the percentage of positively stained area, representing the expression level of each cytokine.

#### 2.4.3 Prognostic evaluation

Prognosis was assessed based on liver function status 6 months after initiation of treatment (De Valle et al., 2006) and classified into four categories: (1) Cured: Complete resolution or significant improvement of clinical symptoms and signs, with normalization of ALT, AST, and other key liver function indices; (2) Improved: Major symptoms were effectively controlled, and serum biochemical markers decreased by more than 50% from baseline; (3) Unresolved: No significant clinical or biochemical improvement, laboratory parameters fluctuated within subclinical ranges or worsened (including cases who voluntarily discontinued treatment); (4) Deceased: Patients who died from DILI-related complications during follow-up.

Based on this classification, patients were divided into two groups for prognostic analysis: favorable prognosis (cured and improved) and unfavorable prognosis (unresolved and deceased). This grouping was used for subsequent analysis of prognostic risk factors.

### 2.5 Statistical analysis

All statistical analyses were performed using SPSS version 27.0 (IBM Corp, Armonk, NY, USA). Data normality was assessed using the Shapiro–Wilk test, and homogeneity of variance was evaluated using Levene’s test. Normally distributed continuous variables were expressed as mean ± standard deviation (x̄ ± s) and compared using the independent-samples t-test or one-way analysis of variance (ANOVA), followed by Bonferroni *post hoc* correction for multiple comparisons when appropriate. Non-normally distributed data were presented as median (interquartile range, IQR) and analyzed using the Mann–Whitney U test or Kruskal–Wallis H test. Categorical variables were compared using the chi-square test or Fisher’s exact test.

Correlations between variables were evaluated using Spearman’s rank correlation analysis. Variables with a *P* value < 0.10 in univariate logistic regression analysis were entered into a multivariate logistic regression model to identify independent prognostic factors. Predictive performance was assessed using receiver operating characteristic (ROC) curve analysis, and the area under the curve (AUC) was calculated. All statistical tests were two-sided, and a P value < 0.05 was considered statistically significant.

## 3 Results

### 3.1 General clinical characteristics of patients with DILI

The baseline characteristics of the 40 patients with DILI are summarized in Table 1. Among them, 90.0% (n = 36) were female and 10.0% (n = 4) were male. The mean age was 50.0 years, and the average body mass index (BMI) was 22.7 kg/m^2^. As shown in Table 2, the most commonly suspected causative agents were traditional Chinese herbal decoctions or proprietary Chinese medicines (47.5%), followed by nonsteroidal anti-inflammatory drugs (22.5%), antimicrobial agents (12.5%), dietary supplements (10.0%), and other drugs (7.5%). The main clinical manifestations included scleral and skin jaundice (62.5%), fatigue (37.5%), and abdominal discomfort (20.0%). Based on the R-value classification, 17 cases (42.5%) were cholestatic type, 16 cases (40.0%) hepatocellular type, and 7 cases (17.5%) mixed type. Following withdrawal of the suspected causative agent, patients received individualized treatment according to their clinical condition. Supportive therapies, including hepatoprotective agents, enzyme-lowering treatments, choleretic therapies, and corticosteroids when clinically indicated, were administered at the discretion of the treating physicians. Upon discharge, 10 patients (25.0%) were cured, 21 (52.5%) showed improvement, and 9 (22.5%) remained unresolved. No cases of acute liver failure, liver transplantation, or death were recorded during the study period. Baseline demographic characteristics of the DILI and healthy control groups are summarized in Supplementary Table S1. Compared with the healthy controls, the DILI group showed a significantly higher proportion of females (90.0% vs. 30.0%, *P* < 0.001) and was significantly older (50.0 ± 9.0 vs. 42.5 ± 6.1 years, *P* < 0.001).

**TABLE 1:** General Characteristics of 40 Patients with Drug-Induced Liver Injury (DILI).

Clinical data	Statistical value
Sex	
Female	36 (90.0%)
Male	4 (10.0%)
Age (years)	50.0 ± 9.0
BMI (kg/m²)	22.7 (21.8, 25.0)
Clinical Pattern	
Cholestatic	17 (42.5%)
Hepatocellular	16 (40.0%)
Mixed	7 (17.5%)
History of Liver Injury	
No	30 (75.0%)
Yes	10 (25.0%)
AST (U/L)	240 (113, 426)
ALP (U/L)	165 (122, 203)
ALT (U/L)	273 (100, 539)
GGT (U/L)	246 (134, 339)
CHE (U/L)	6035 ± 2385
ALB (g/L)	38.2 ± 5.2
TBiL (µmol/L)	56 (17, 132)
DBiL (µmol/L)	28 (5, 76)
IBiL (µmol/L)	27 (13, 58)
TC (mmol/L)	4.21 (3.41, 5.03)
TG (mmol/L)	1.94 ± 1.19
HDL (mmol/L)	0.96 ± 0.53
LDL (mmol/L)	2.74 (2.22, 3.25)
INR	1.07 (0.98, 1.17)
WBC (10⁹/L)	5.08 ± 1.85
HB (g/L)	125 (111, 133)
Cr (µmol/L)	57 (44, 63)
CRP (mg/L)	3.5 (1.9, 7.5)
Treatment	
Medication only	29 (72.5%)
Medication + steroids	11 (27.5%)
Prognosis	
Favorable	31 (77.5%)
Unfavorable	9 (22.5%)

Abbreviations: BMI, body mass index; AST, aspartate aminotransferase; ALP, alkaline phosphatase; ALT, alanine aminotransferase; GGT, gamma-glutamyl transferase; CHE, cholinesterase; ALB, albumin; TBiL, total bilirubin; DBiL, direct bilirubin; IBiL, indirect bilirubin; TC, total cholesterol; TG, triglycerides; HDL, high-density lipoprotein cholesterol; LDL, low-density lipoprotein cholesterol; INR, international normalized ratio; WBC, white blood cell count; HB, hemoglobin; Cr, creatinine; CRP, C-reactive protein.

**TABLE 2:** Categories and Proportions of Drugs Causing DILI.

Drug Category	Generic Name or Composition	Number of Cases	Proportion (%)
Traditional Chinese Medicines	Herbal decoctions, Guci Plaster, Lianhua Qingwen Capsules, Licorice Tablets, Relinqing Granules, Yangxue Qingnao Granules, Liuwei Dihuang Pills, Bailing Capsules	19	47.50
NSAIDs	Ibuprofen, Compound Paracetamol Tablets, Acetaminophen	9	22.50
Antibiotics	Metronidazole, Roxithromycin	5	12.50
Antirheumatic Agents	Febuxostat, Benzbromarone, Methotrexate	2	5.00
Health Supplements	Seal oil, etc.	4	10.00
Environmental Toxins	Hair dye	1	2.50

Abbreviations: DILI, drug-induced liver injury; NSAIDs, nonsteroidal anti-inflammatory drugs.

### 3.2 Histopathological characteristics of different DILI subtypes

Among the 40 DILI patients, distinct histopathological features were observed across different injury patterns. The cholestatic subtype was characterized by hepatocellular and canalicular cholestasis, bile pigment accumulation, mild hepatocellular injury, and predominantly microvesicular steatosis. The hepatocellular subtype commonly exhibited interface hepatitis, spotty necrosis, hepatocellular hydropic degeneration, ductular reaction, and mixed inflammatory cell infiltration. The mixed subtype displayed overlapping features of both cholestatic and hepatocellular injury, including moderate-to-severe hepatocellular damage, cholestasis, and mixed inflammatory infiltration.

### 3.3 Cytokine expression levels and their association with the pathological features of DILI

**FIGURE 2:**
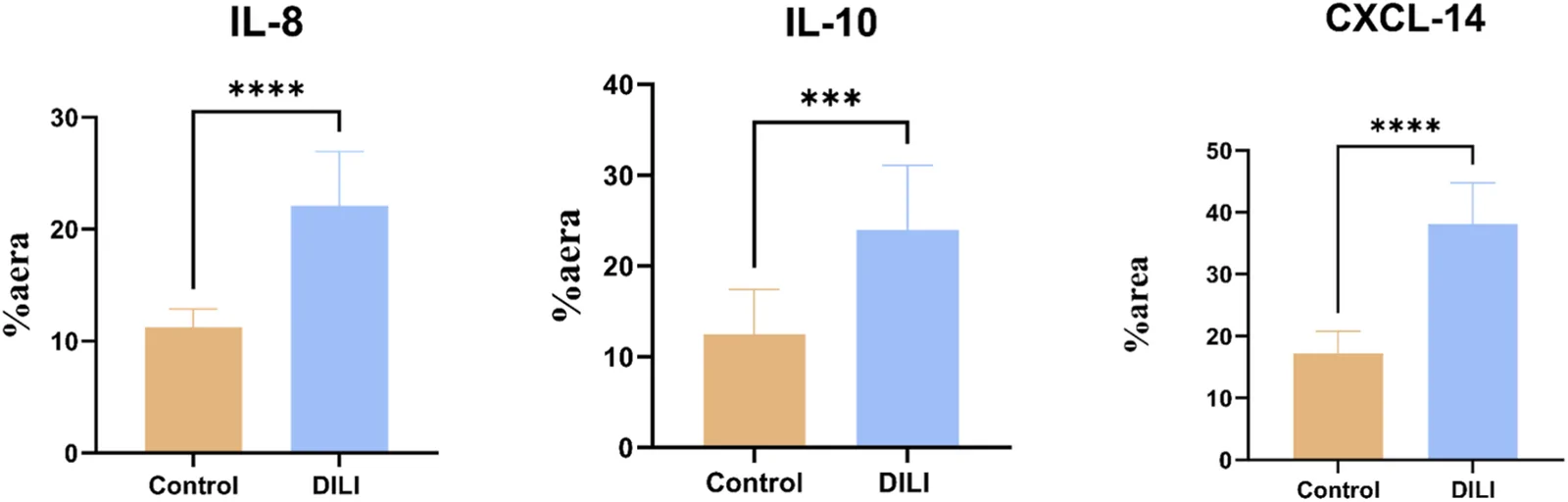
Comparison of IL-8, IL-10, and CXCL-14 Levels Between DILI Patients and Healthy Controls. Representative immunohistochemical staining images and quantitative analysis comparing the normal control group (n = 10) and the DILI group (n = 40). Original magnification: ×400. Scale bar = 20 μm.

Cytokine expression in liver tissues was assessed in 40 patients with DILI and 10 healthy controls. Compared with the healthy control group, all three cytokines—IL-8, IL-10, and CXCL-14—showed differential expression in the DILI group (Table 3; Figure 2).

**TABLE 3:** Comparison of IL-8, IL-10, and CXCL-14 Levels Between DILI and Healthy control groups.

Group	IL-8 (Mean ± SD)	IL-10 (Mean ± SD)	CXCL-14 (Mean ± SD)
DILI Group (n = 40)	22.08 ± 4.89	23.98 ± 7.12	38.10 ± 6.64
Healthy control Group (n = 10)	11.25 ± 1.62	12.49 ± 4.96	17.24 ± 3.55
*t-value*	5.744	4.029	7.983
*P*	< 0.0001	0.003	< 0.001

Abbreviations: IL-8, interleukin-8; IL-10, interleukin-10; CXCL-14, C-X-C motif chemokine ligand 14.

**FIGURE 3:**
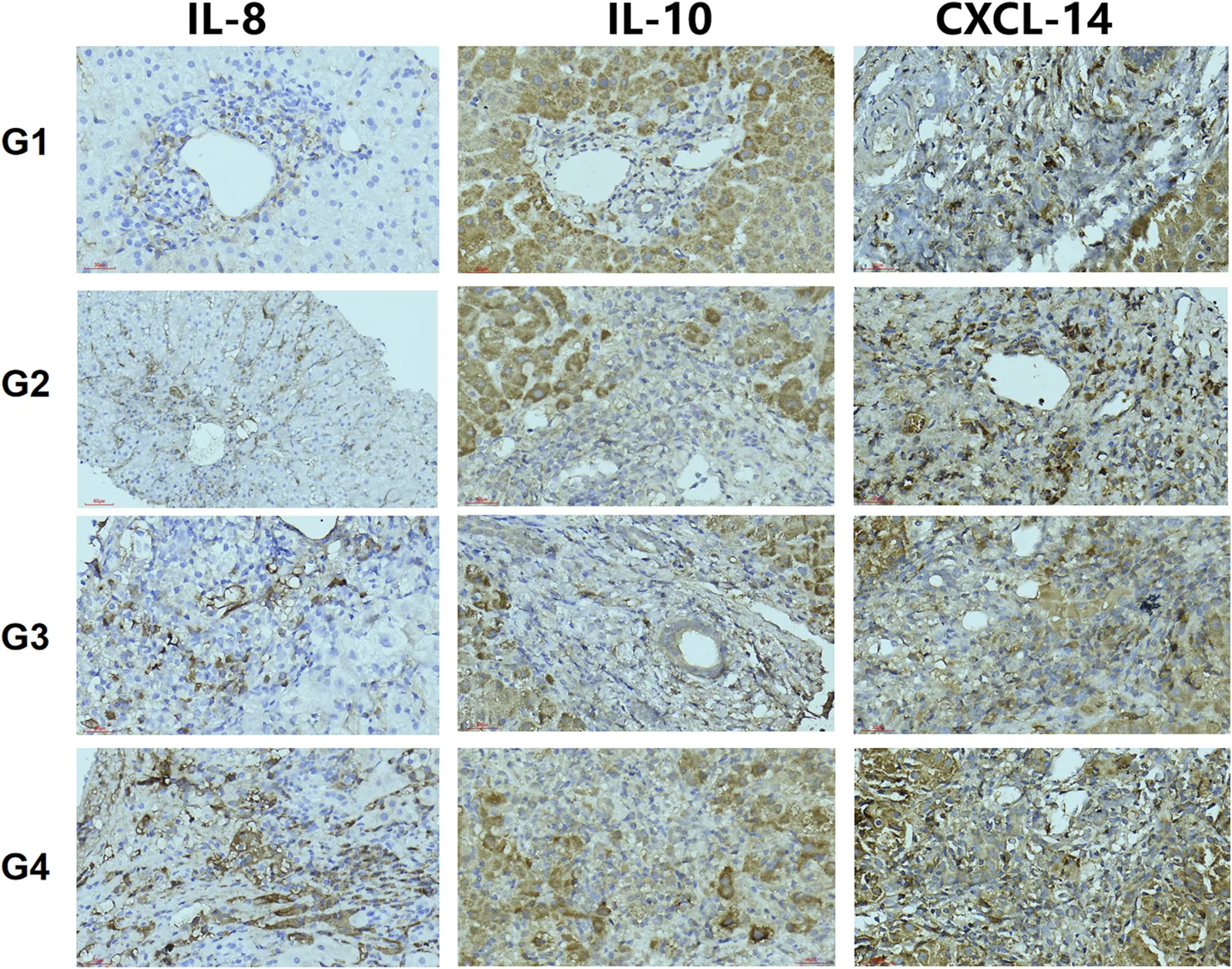
Representative immunohistochemical staining images of IL-8, IL-10, and CXCL-14 across pathological inflammation grades in DILI liver tissue. Brown staining indicates positive expression of the target cytokines. Inflammation grade subgroup sizes were G1 (n = 10), G2 (n = 10), G3 (n = 10), and G4 (n = 10). Representative images from each subgroup are shown. Images were acquired using light microscopy at an original magnification of ×400. Scale bar = 20 μm. Quantitative analysis of cytokine expression across inflammation grades is presented in Table 4.

Based on portal area inflammatory infiltration and lobular activity, the pathological inflammation grades of DILI patients were classified into four subgroups: G1 (n = 10), G2 (n = 10), G3 (n = 10), and G4 (n = 10) (Figure 3). The expression levels of the three cytokines were compared among these groups (Table 4). IL-8 and CXCL-14 showed significant differences across inflammation grades, with expression increasing alongside inflammation severity (*P* < 0.05). In contrast, IL-10 expression differences did not reach statistical significance. Spearman correlation analysis further confirmed that IL-8 (*r* = 0.53, *P* < 0.01), IL-10 (*r* = 0.43, *P* < 0.01), and CXCL-14 (*r* = 0.37, *P* < 0.05) were all positively correlated with inflammation grade.

**TABLE 4:** Comparison of IL-8, IL-10, and CXCL-14 Levels Among Patients With Different Degrees of Inflammation (G1–G4).

Cytokine	G1 (n = 10)	G2 (n = 10)	G3 (n = 10)	G4(n=10)	*P*
IL-8 (Mean ± SD)	18.50 ± 4.56	22.26 ± 3.72	23.00 ± 4.20	25.89±4.65	0.012
IL-10 (Mean ± SD)	18.53 ± 7.27	23.63 ± 7.52	25.93 ± 5.97	28.74±3.66	0.062
CXCL-14 (Mean ± SD)	31.56 ± 8.67	39.59 ± 6.02	38.50 ± 4.79	42.19±2.7	0.031

Abbreviations: IL-8, interleukin-8; IL-10, interleukin-10; CXCL-14, C-X-C motif chemokine ligand 14.

**FIGURE 4:**
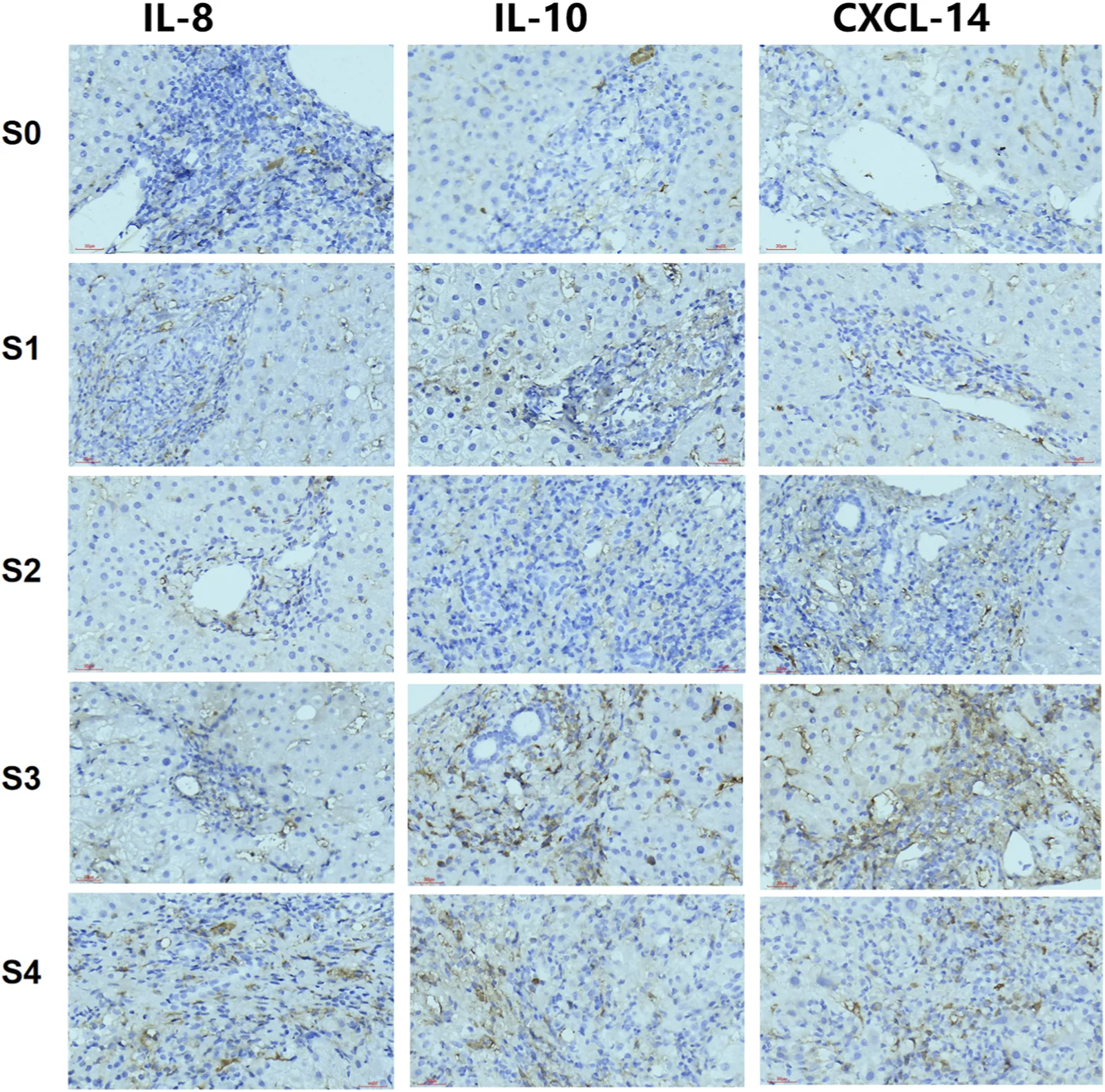
Representative immunohistochemical staining images of IL-8, IL-10, and CXCL-14 across fibrosis stages in DILI liver tissue. Brown staining indicates positive expression of the target cytokines. Fibrosis stage subgroup sizes were S0 (n = 7), S1 (n = 13), S2 (n = 9), S3 (n = 8), and S4 (n = 3). Representative images from each subgroup are shown. Images were acquired using light microscopy at an original magnification of ×400. Scale bar = 20 μm. Quantitative analysis of cytokine expression across fibrosis stages is presented in Table 5.

Patients were also stratified into five subgroups according to liver fibrosis stage: S0 (n = 7), S1 (n = 13), S2 (n = 9), S3 (n = 8), and S4 (n = 3) (Figure 4). Cytokine expression levels were compared across fibrosis stages (Table 5). IL-8 expression increased progressively with fibrosis severity, with statistically significant differences observed among groups (*P* < 0.05). However, CXCL-14 and IL-10 did not exhibit significant differences between fibrosis stages. Spearman correlation analysis showed that IL-8 (*r* = 0.36, *P* = 0.037) and CXCL-14 (*r* = 0.38, *P* = 0.036) were positively correlated with fibrosis stage, whereas IL-10 was not significantly correlated (*r* = 0.33, *P* = 0.069).

**TABLE 5:** Comparison of IL-8, IL-10, and CXCL-14 Levels Among Patients With Different Fibrosis Stages (S0–S4).

Cytokine	S0 (n = 7)	S1 (n = 13)	S2 (n = 9)	S3 (n = 8)	S4 (n = 3)	P
IL-8	17.21 ± 2.58	23.25 ± 5.13	23.68 ± 3.82	23.99 ± 5.58	20.58 ± 4.19	0.038
IL-10	18.05 ± 7.31	23.11 ± 8.35	26.54 ± 6.28	26.25 ± 4.93	25.96 ± 2.92	0.252
CXCL-14	31.12 ± 9.62	38.09 ± 6.51	40.10 ± 4.13	39.72 ± 3.91	43.45 ± 0.78	0.084

Abbreviations: IL-8, interleukin-8; IL-10, interleukin-10; CXCL-14, C-X-C motif chemokine ligand 14.

### 3.4 Correlation between cytokines and liver function parameters

**FIGURE 5:**
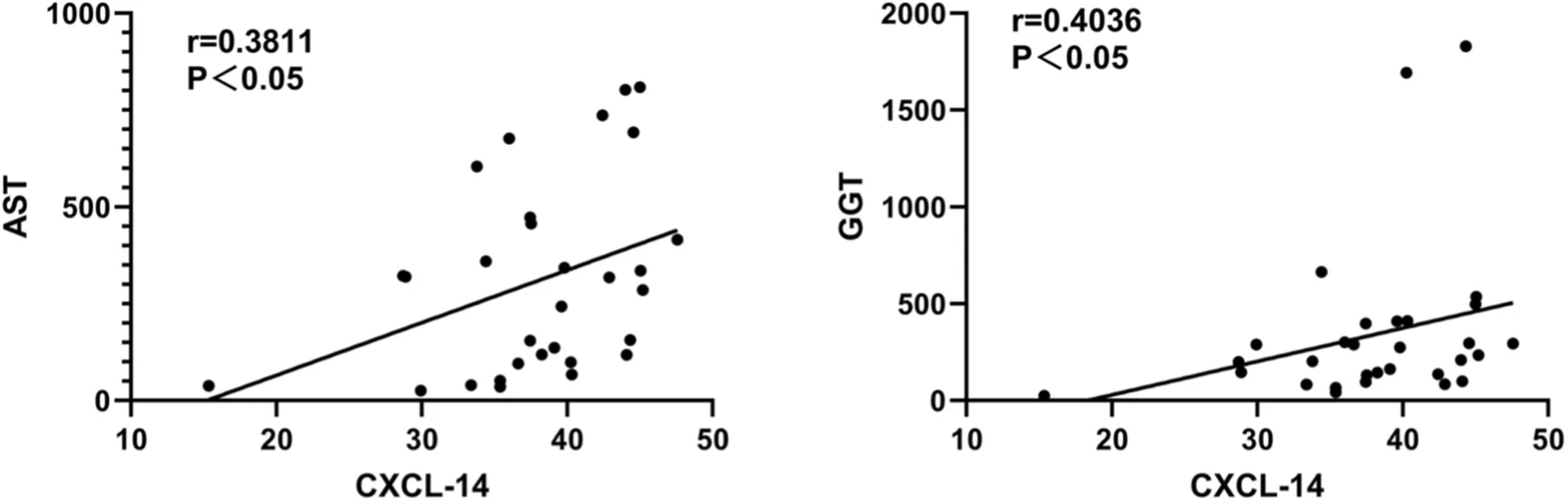
Positive Correlation Between CXCL-14 and AST and GGT. Scatter plots showing the correlations between hepatic CXCL-14 expression and serum AST and GGT levels. Correlation coefficients were calculated using Spearman’s rank correlation analysis. Two patients exhibited relatively high GGT values; these were verified against the original clinical records and retained in the analysis.

Spearman correlation analysis was performed to assess the relationship between cytokine levels and liver function parameters in the DILI group. The results revealed that CXCL-14 was positively correlated with aspartate aminotransferase (AST) and gamma-glutamyl transpeptidase (GGT), with correlation coefficients r = 0.381 (*P* = 0.038) and *r* = 0.404 (*P* = 0.027), respectively (Table 6; Figure 5). Two patients exhibited relatively high GGT values. These values were verified against the original clinical records and were retained in the analysis because they represented true clinical observations rather than data entry or measurement errors.

**TABLE 6:** Correlation Between Cytokine Levels and Liver Function Parameters.

Liver function parameter	IL-8 (r)	P	IL-10 (r)	P	CXCL-14 (r)	P
AST	0.284	0.104	0.108	0.564	0.381	0.038
ALP	0.055	0.757	–0.024	0.897	0.337	0.069
ALT	0.116	0.514	0.069	0.712	0.295	0.114
GGT	0.170	0.338	0.136	0.465	0.404	0.027
CHE	–0.338	0.051	–0.118	0.527	–0.143	0.452
ALB	–0.326	0.060	–0.255	0.166	–0.154	0.416
TBiL	0.137	0.439	0.260	0.158	0.348	0.059
DBiL	0.166	0.348	0.247	0.180	0.349	0.059
IBiL	0.069	0.698	0.276	0.133	0.300	0.108

Abbreviations: AST, aspartate aminotransferase; ALP, alkaline phosphatase; ALT, alanine aminotransferase; GGT, gamma-glutamyl transferase; CHE, cholinesterase; ALB, albumin; TBiL, total bilirubin; DBiL, direct bilirubin; IBiL, indirect bilirubin.

### 3.5 Prognostic evaluation and biomarker analysis in DILI patients

**FIGURE 6:**
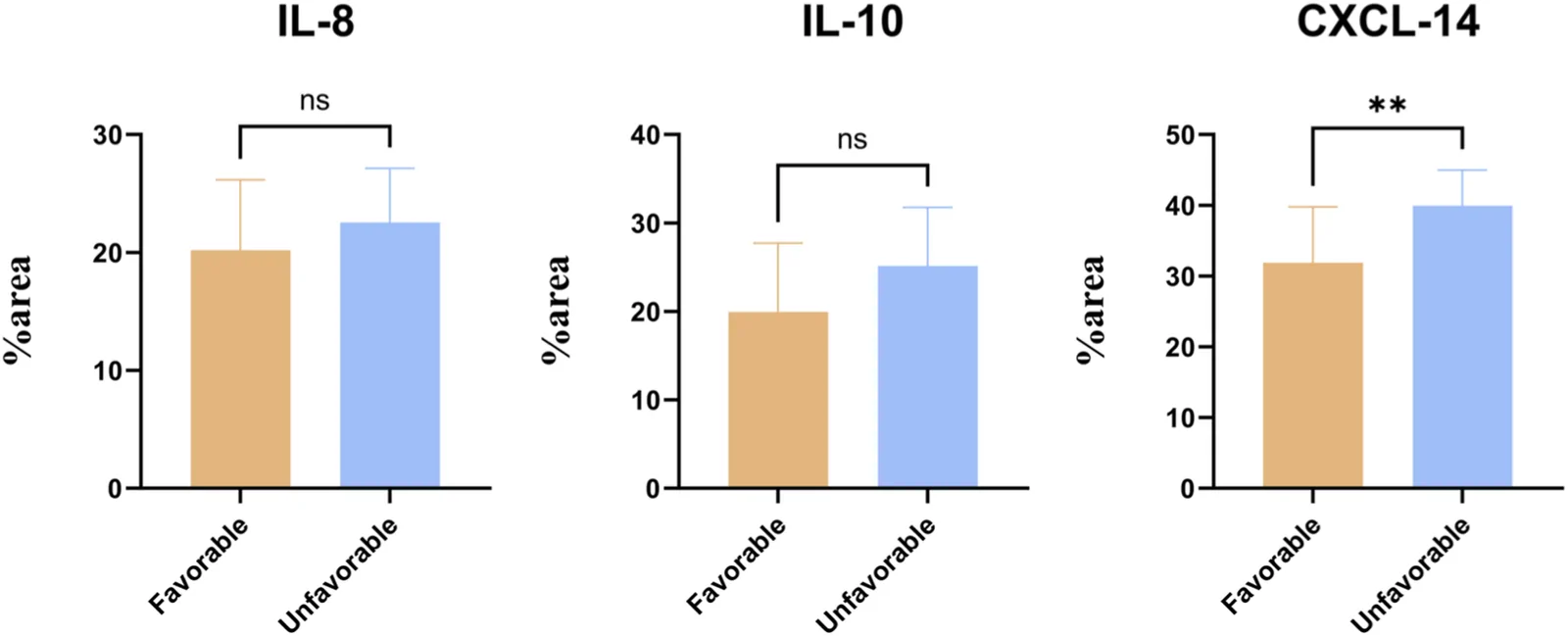
Comparison of IL-8, IL-10, and CXCL-14 Levels Between Patients With Different Prognoses. Comparison of hepatic IL-8, IL-10, and CXCL-14 expression between patients with favorable prognosis (n = 31) and unfavorable prognosis (n = 9). ROC curve analysis of CXCL-14 for predicting clinical prognosis is also presented.

Based on clinical outcomes assessed 6 months after treatment initiation, patients were categorized into a favorable prognosis group (n = 31) and an unfavorable prognosis group (n = 9). Among the three cytokines analyzed, CXCL-14 levels showed a significant difference between the two groups (Table 7; Figure 6).

**TABLE 7:** Comparison of IL-8, IL-10, and CXCL-14 Levels Between Patients With Favorable and Unfavorable Prognosis.

Group	IL-8 (Mean ± SD)	IL-10 (Mean ± SD)	CXCL-14 (Mean ± SD)
Favorable Prognosis (n = 31)	20.20 ± 5.97	19.95 ± 7.80	31.88 ± 7.92
Unfavorable Prognosis (n = 9)	22.56 ± 4.58	25.15 ± 6.63	39.99 ± 4.99
*t-value*	1.145	1.757	3.268
P	0.260	0.089	0.002

Abbreviations: IL-8, interleukin-8; IL-10, interleukin-10; CXCL-14, C-X-C motif chemokine ligand 14.

Univariate logistic regression analysis identified CXCL-14, GGT, and INR as variables associated with clinical prognosis (Table 8). Variables with *P* < 0.10 in the univariate analysis were subsequently entered into a multivariate logistic regression model (Table 9), The final multivariate logistic regression model identified CXCL-14 as an independent risk factor for unfavorable prognosis in patients with DILI (OR = 1.769, 95% CI: 1.062–2.947, *P* = 0.029), indicating that higher hepatic CXCL-14 expression was significantly associated with an increased risk of adverse clinical outcomes.

**TABLE 8:** Univariate Logistic Regression Analysis of Prognostic Factors in DILI Patients.

Variable	Category	B	S.E.	Wald	P	OR	95% CI
Sex	Male (Ref)	—	—	—	—	—	—
	Female	–1.253	1.077	1.352	0.245	0.286	0.035–2.360
Age	—	–0.006	0.040	0.022	0.881	0.994	0.919–1.075
Clinical Type	Cholestatic (Ref)	—	—	—	0.760	—	—
	Hepatocellular	0.041	0.992	0.002	0.967	1.042	0.149–7.275
	Mixed	–0.550	1.054	0.273	0.602	0.577	0.073–4.550
Liver Injury History	Yes	–0.342	0.814	0.177	0.674	0.710	0.144–3.501
	No (Ref)	—	—	—	—	—	—
BMI	—	0.027	0.105	0.068	0.795	1.028	0.836–1.263
AST	—	0.002	0.001	1.303	0.254	1.002	0.998–1.004
ALP	—	–0.003	0.004	0.536	0.464	0.997	0.990–1.004
ALT	—	0.001	0.001	1.000	0.317	1.001	0.999–1.003
GGT	—	0.013	0.005	7.252	0.007	1.013	1.003–1.022
CHE	—	0.024	0.038	0.617	0.432	1.024	0.951–1.103
ALB	—	0.009	0.071	0.015	0.904	1.009	0.877–1.160
TBiL	—	–0.001	0.002	0.214	0.664	0.999	0.996–1.002
DBiL	—	0.001	0.004	0.014	0.906	1.001	0.992–1.009
IBiL	—	0.000	0.008	0.004	0.951	1.000	0.984–1.015
INR	—	2.487	1.159	4.60	0.032	12.030	1.240 – 116.600
WBC	—	0.117	0.197	0.353	0.553	1.124	0.764–1.654
HB	—	0.010	0.016	0.429	0.513	1.010	0.980–1.042
Cr	—	0.022	0.015	2.304	0.129	1.023	0.994–1.052
CRP	—	0.148	0.080	3.409	0.065	1.160	0.991–1.357
IL-8	—	-0.039	0.096	0.169	0.681	0.961	0.797 – 1.159
IL-10	—	-0.100	0.093	1.171	0.279	0.905	0.755–1.085
CXCL-14	—	0.241	0.110	4.799	0.028	1.273	1.026 – 1.579

Abbreviations: BMI, body mass index; AST, aspartate aminotransferase; ALP, alkaline phosphatase; ALT, alanine aminotransferase; GGT, gamma-glutamyl transferase; CHE, cholinesterase; ALB, albumin; TBiL, total bilirubin; DBiL, direct bilirubin; IBiL, indirect bilirubin; INR, international normalized ratio; WBC, white blood cell count; HB, hemoglobin; Cr, creatinine; CRP, C-reactive protein; IL-8, interleukin-8; IL-10, interleukin-10; CXCL-14, C-X-C motif chemokine ligand 14; OR, odds ratio; CI, confidence interval.

**TABLE 9:** Multivariate Logistic Regression Analysis of Prognostic Factors in DILI Patients.

Variable	B	S.E.	Wald	P	OR	95% CI
GGT	−0.278	0.224	1.54	0.214	0.757	0.523–1.097
INR	2.318	1.229	3.56	0.059	10.149	0.904–113.626
CXCL-14	0.570	0.262	4.813	0.029	1.769	1.062–2.947

Abbreviations: GGT, gamma-glutamyl transferase; INR, international normalized ratio; CXCL-14, C-X-C motif chemokine ligand 14; OR, odds ratio; CI, confidence interval.

Receiver operating characteristic (ROC) curve analysis demonstrated good predictive performance of CXCL-14 for clinical prognosis, with an area under the curve (AUC) of 0.848 (95% CI: 0.708–0.988, *P* = 0.003), a sensitivity of 69.6%, and a specificity of 85.7%, suggesting that CXCL-14 may serve as a valuable prognostic biomarker for DILI.

### 3.6 Cellular sources of cytokines in DILI liver tissue

**FIGURE 7:**
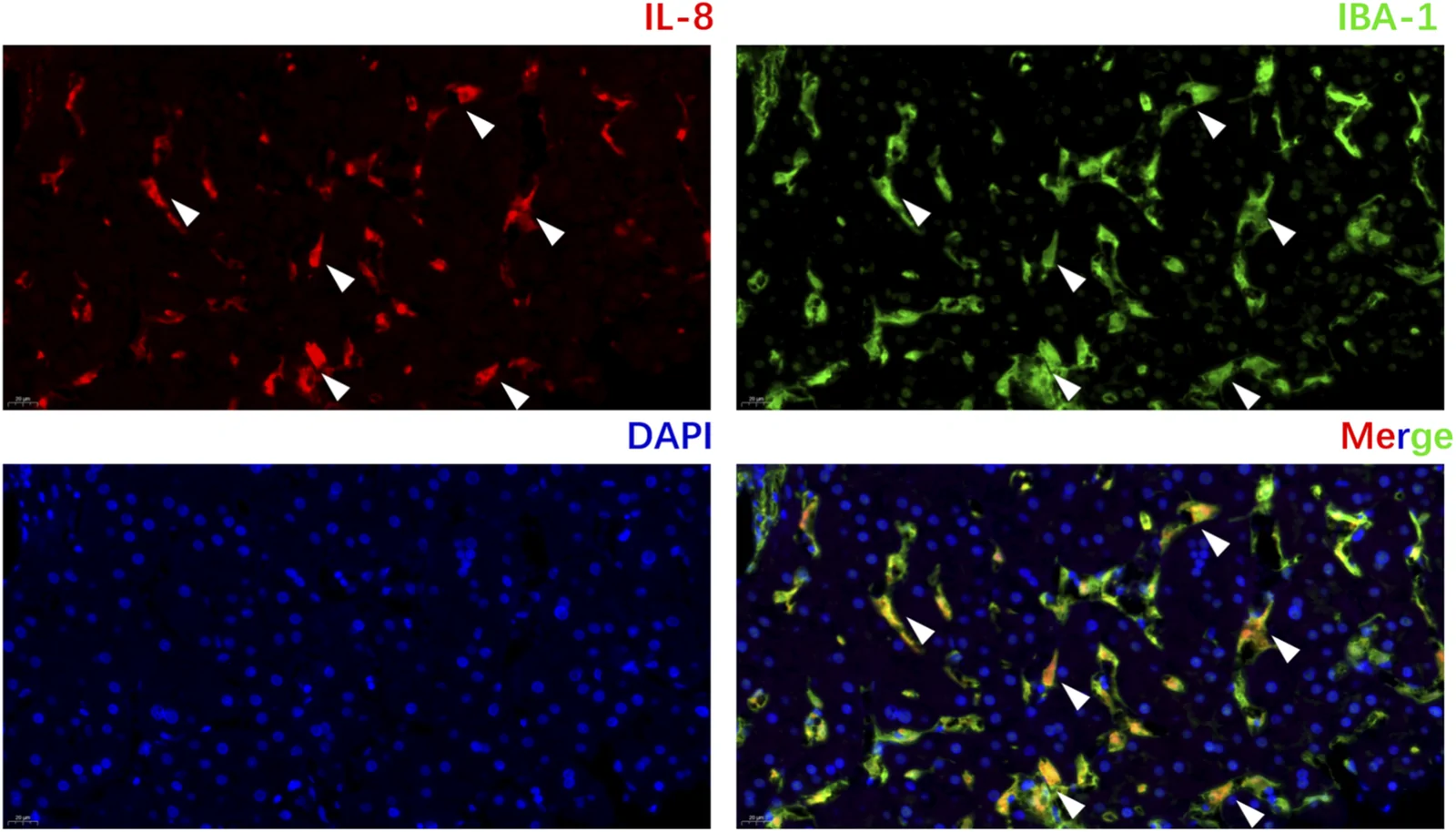
Colocalization of IL-8 With the Macrophage Marker IBA-1 in DILI Liver Tissue. Representative multiplex immunofluorescence images showing the colocalization of IL-8 with the macrophage marker IBA-1 in DILI liver tissue. Nuclei were counterstained with DAPI. Original magnification: ×400. Scale bar = 20 μm.

To further identify the cellular sources of IL-8, IL-10, and CXCL-14 in liver tissue from DILI patients, multiplex immunofluorescence staining was performed. The results demonstrated clear colocalization of IL-8 signals with the macrophage marker IBA-1 (Figure 7), indicating a close association between IL-8 expression and hepatic macrophages. This suggests that macrophages are the predominant cellular source of IL-8 during the progression of DILI, providing a critical cellular target for understanding the inflammatory regulatory mechanisms involved in DILI. However, the precise cellular sources of IL-10 and CXCL-14 in DILI liver tissue remain unclear.

## 4 Discussion

This study, based on 40 biopsy-confirmed DILI patients, quantitatively analyzed the hepatic expression of three inflammation-related cytokines (IL-8, IL-10, and CXCL-14) and explored their potential roles in the pathogenesis and prognostic evaluation of DILI, in conjunction with pathological grading, liver function parameters, and clinical outcomes.

Firstly, we found a higher incidence of DILI among middle-aged and elderly Chinese women, with a strong association with traditional Chinese medicine (TCM) usage. This finding is consistent with several domestic studies (Amacher, 2014), highlighting the urgent need for standardized monitoring and regulatory oversight of TCM safety.

Secondly, the histopathological features of DILI are markedly heterogeneous, encompassing a wide spectrum from acute hepatitis to chronic liver fibrosis (Apel et al., 2020; Lai et al., 2012), which poses a significant challenge to accurate diagnosis. In our cohort, The cholestatic subtype was characterized by hepatocellular and canalicular cholestasis with bile pigment accumulation, whereas the hepatocellular subtype commonly exhibited interface hepatitis, spotty necrosis, hepatocellular hydropic degeneration, ductular reaction, and mixed inflammatory cell infiltration. Mixed-type cases displayed overlapping features of both cholestatic and hepatocellular injury, reflecting the marked pathological heterogeneity of DILI. These findings closely align with the pathological classification proposed by the U.S. Drug-Induced Liver Injury Network (DILIN) (Kleiner et al., 2014), affirming the unique value of histopathology in elucidating injury mechanisms and assessing disease activity. However, limited by the sample size (n = 40), our study did not yield statistically significant results in quantitative assessments such as bile duct reaction. Future multicenter studies with larger cohorts are warranted to develop standardized histological evaluation criteria, particularly for parameters such as steatosis quantification and bile duct reaction grading.

In terms of cytokine expression, IL-8 and CXCL-14 were significantly upregulated in DILI patients and positively correlated with the degree of hepatic inflammation. IL-8, a potent neutrophil chemoattractant, has been well-established as a pro-inflammatory mediator in various liver diseases (Ma et al., 2022). Our results further underscore its role in amplifying inflammation in drug-induced hepatitis. The marked increase in CXCL-14 is of particular interest. Despite limited prior research, its strong association with fibrosis severity in this study suggests a possible involvement in chronic tissue remodeling and fibrogenesis (Li et al., 2011). While IL-10 was also elevated in DILI patients, its expression did not correlate with fibrosis, implying that its anti-inflammatory role in DILI may be limited and likely reflects a nonspecific immunoregulatory feedback (Bourdi et al., 2002). This finding is partly consistent with previous studies reporting increased IL-10 expression during liver injury as a compensatory anti-inflammatory response aimed at limiting excessive immune-mediated tissue damage. Unlike pro-inflammatory cytokines such as IL-8, IL-10 may reflect activation of regulatory pathways rather than ongoing disease activity. The absence of significant associations with fibrosis stage or clinical prognosis in our cohort suggests that IL-10 may have limited value as a standalone biomarker for disease severity, although its dynamic role during different stages of DILI progression warrants further investigation. These observations point to the need for further studies aimed at establishing a dynamic monitoring framework for cytokine regulation in DILI.

In the prognostic analysis, CXCL-14 was identified as an independent risk factor for poor outcomes in DILI patients through multivariate logistic regression. ROC curve analysis yielded an AUC of 0.848, indicating good prognostic performance for predicting clinical outcomes in patients with DILI. These findings suggest that CXCL-14 may serve as a promising prognostic biomarker and may have potential clinical value in DILI risk assessment and individualized therapeutic management. As a novel chemokine, CXCL-14 may contribute to fibrosis progression by regulating immune cell recruitment, amplifying inflammatory responses, and activating hepatic stellate cells. However, its specific signaling pathways, target cell types, and regulatory networks in DILI remain poorly understood and warrant further investigation through molecular and functional studies. Whether CXCL-14 blockade has therapeutic potential also remains to be confirmed in pharmacological experiments.

Multiplex immunofluorescence staining in this study revealed marked colocalization of IL-8 with the macrophage marker IBA-1 in DILI liver tissues, suggesting that macrophages may be the primary source of IL-8. This finding is consistent with previous studies indicating macrophage-dominated inflammatory responses in liver injury (Gao et al., 2008). In contrast, the cellular localization of IL-10 and CXCL-14 remained inconclusive, possibly due to their low expression levels, widespread tissue distribution, or co-expression in multiple cell types. Some studies have shown that IL-10 may be produced by multiple immune and hepatic cell populations under inflammatory conditions (Sun and Tian, 2010), while CXCL-14 may originate from hepatocytes, stellate cells, or endothelial cells and may play a role in maintaining tissue homeostasis and mediating fibrogenesis (Tsuchiya and Kaisho, 2020). Given the limitations of conventional immunofluorescence in multiplexing and spatial resolution, future studies should consider the use of high-throughput technologies such as spatial transcriptomics and single-cell sequencing to clarify the specific sources and functional roles of these cytokines.

This study has several novel contributions: (1) it is the first to systematically assess the expression profile and clinical relevance of CXCL-14 in liver tissues of DILI patients; (2) it proposes CXCL-14 as a potential independent biomarker for long-term prognosis in DILI; and (3) it integrates pathological grading, quantitative immunohistochemistry, and clinical follow-up data to establish a comprehensive “mechanism–phenotype–prognosis” analytical framework.

However, the study also has some limitations. First, the sample size was relatively small, particularly in the unfavorable prognosis subgroup, which limited the statistical power of the prognostic analyses and prevented further subtype analysis of cytokine expression across different pathological patterns. Therefore, the multivariate logistic regression results should be interpreted with caution and require validation in larger independent cohorts. Second, as a single-center retrospective study, it is inherently subject to potential selection bias. Third, although all cases underwent retrospective causality assessment using the RUCAM score and achieved scores consistent with a probable diagnosis of DILI, the retrospective single-center design may still introduce a degree of diagnostic uncertainty. Fourth, the healthy control cohort was relatively small and differed significantly from the DILI cohort in both age and sex distribution. Therefore, residual confounding by these demographic differences cannot be completely excluded when interpreting the observed differences in hepatic cytokine expression. Future studies with larger age- and sex-matched control cohorts are warranted. Finally, mechanistic studies were lacking, and it remains unclear whether CXCL-14 upregulation represents a causal factor or a reactive response. In addition, detailed treatment-related information, including specific medications, dosages, and corticosteroid regimens, was not systematically recorded and therefore could not be evaluated in the present study.

## 5 Conclusion

1. IL-8, IL-10, and CXCL-14 were all aberrantly expressed in liver tissues of DILI patients. Among them, IL-8 expression was closely associated with the severity of hepatic inflammation and fibrosis, indicating its potential as a biomarker for assessing disease severity.
2. CXCL-14 was identified as an independent risk factor associated with poor prognosis in DILI and demonstrated promising prognostic performance in this cohort. Future studies with larger sample sizes and multicenter validation are needed to further confirm its prognostic value and to elucidate the biological mechanisms underlying its involvement in DILI.

## Data Availability

The raw data supporting the conclusions of this article will be made available by the authors, without undue reservation.
