# Supplementary material for: Hepatic expression of IL-8, IL-10, and CXCL-14 in drug-induced liver injury: correlation with histopathology and prognostic value
Source: Front Pharmacol. 2026 Aug 12;17:1882357. doi: 10.3389/fphar.2026.1882357 (PMC13506696; doi:10.3389/fphar.2026.1882357)
Supplement: Supplementary file 1 [file Table1.doc]

**Supplementary Table S1. Baseline demographic and clinical characteristics of the DILI and healthy control groups.**

| **Variable** | **DILI Group (n = 40)** | **Control Group (n = 10)** | ***P*** |
| --- | --- | --- | --- |
| Sex, n (%) |  |  |  |
| Female | 36 (90.0%) | 3(30.0%) | <0.001 |
| Male | 4 (10.0%) | 7(70.0%) |  |
| Age (years) | 50.0 ± 9.0 | 42.5 ± 6.1 | <0.001 |
| BMI (kg/m²) | 22.7 (21.8, 25.0) | 21.4 (19.4, 22.3) | 0.002 |
| AST (U/L) | 240 (113, 426) | 22 (16, 48.1) | <0.001 |
| ALP (U/L) | 165 (122, 203) | 124 (77, 134) | 0.481 |
| ALT (U/L) | 273 (100, 539) | 168 (32, 214) | 0.528 |
| GGT (U/L) | 246 (134, 339) | 50.6 (41.3, 57.1) | 0.004 |
| CHE (U/L) | 6035 ± 2385 | 6678 ± 2623 | 0.458 |
| ALB (g/L) | 38.2 ± 5.2 | 39.1 ± 6.0 | 0.842 |
| TBiL (µmol/L) | 56 (17, 132) | 14.7 (12.5, 18.9) | 0.001 |
| DBiL (µmol/L) | 28 (5, 76) | 4.6 (2.6, 6.5) | 0.003 |
| IBiL (µmol/L) | 27 (13, 58) | 13.7 (9.6, 17.4) | 0.006 |
| TC (mmol/L) | 4.21 (3.41, 5.03) | 3.82 (3.49, 4.89) | 0.934 |
| TG (mmol/L) | 1.94 ± 1.19 | 2.09 ± 1.31 | 0.308 |
| HDL (mmol/L) | 0.96 ± 0.53 | 1.16 ± 0.57 | 0.173 |
| LDL (mmol/L) | 2.74 (2.22, 3.25) | 2.16 (1.65, 3.87) | 0.356 |
| INR | 1.07 (0.98, 1.17) | 1.10 (1.00, 1.23) | 0.382 |
| WBC (10⁹/L) | 5.08 ± 1.85 | 4.93 ± 1.59 | 0.371 |
| HB (g/L) | 125 (111, 133) | 134 (127, 166) | 0.912 |
| Cr (µmol/L) | 57 (44, 63) | 48 (34, 76) | 0.364 |
| CRP (mg/L) | 3.5 (1.9, 7.5) | 7.79 (5.61, 9.87) | 0.319 |

Data are presented as mean ± standard deviation (SD), median (interquartile range), or n (%). Continuous variables were compared using Student's t-test or the Mann–Whitney U test, as appropriate. Categorical variables were compared using the χ² test or Fisher's exact test, as appropriate.
